# Evaluation of in vitro chondrocytic differentiation: A stem cell research initiative at the King Abdulaziz University, Kingdom of Saudi Arabia

**DOI:** 10.6026/97320630014053

**Published:** 2018-02-28

**Authors:** Aisha Al-Yamani, Gauthaman Kalamegam, Farid Ahmed, Mohammed Abbas, Khalid Hussein Wali Sait, Nisreen Anfinan, Mohammad Khalid Al-Wasiyah, Etimad A Huwait, Mamdouh Gari, Mohammed Al-Qahtani

**Affiliations:** 1 Department of Biochemistry, Faculty of Science, King Abdulaziz University, Jeddah, Kingdom of Saudi Arabia; 2 Stem Cell Unit, Centre of Excellence in Genomic Medicine Research, King Abdulaziz University, Jeddah, Saudi Arabia; 3 Sheikh Salem Bin Mahfouz Scientific Chair for Treatment of Osteoarthritis by Stem Cells, King Abdulaziz University, Jeddah, Saudi Arabia; 4 Department of Orthopaedic Surgery, Faculty of Medicine, King Abdulaziz University, Jeddah, Saudi Arabia; 5 Department of Obstetrics and Gynaecology, Faculty of Medicine, King Abdulaziz University, Jeddah, Saudi Arabia; 6 Department of Medical Laboratory Technology, Faculty of Applied Medical Sciences, King Abdulaziz University, Jeddah, Kingdom of Saudi Arabia

**Keywords:** hWJSCs, Differentiation, in vitro

## Abstract

Mesenchymal stem cells (MSCs) from various sources have been used in cartilage differentiation with variable success. Therefore, it is of interest to evaluate the in vitro differentiation potential of the hWJSCs derived from the human umbilical cords into chondrocytes at the stem cell research facility at the King Abdulaziz University. hWJSCs are an attractive choice for tissue engineering and regenerative medical applications including cartilage regeneration. We evaluated the hWJSCs using classical histological and cartilage related gene expression studies. Some of the known parameters were re-examined for consistency at the current laboratory conditions. Early passages (P1-P4) showed short fibroblastic morphology and high expression of MSC related surface markers namely CD29 (99.9%), CD44 (97.8%), CD73 (99.6%), CD90 (95.1%) and CD105 (98.9%). MTT assay showed time dependent increase in hWJSCs proliferation by 61.06% and 206.31% at 48h and 72h respectively. Toluidine blue histology showed that hWJSCs were successfully differentiated into chondrocytes in chondrocytic differentiation medium for 21 days. Differentiated hWJSCs also showed significantly increased expression of collagen type II, aggrecan and SOX9 compared to the undifferentiated control. It should be noted that the determination of the average cell yield, the population doubling time and histological staining wtih alcian blue and/or safronin O is required in future studies for improved evaluation of differentiation. Painless derivation, abundance of stem cells that are hypo-immunogenic and safety issues makes this method advantages to MSCs derived from other sources.

## Background

Millions of people worldwide suffer from osteoarthritis (OA), a degenerative disease of the joints that is characterized by pain, swelling, stiffness, narrowing of joint space, osteophyte formation and articular cartilage degeneration [1]. OA is a major medical, social and economic burden and is projected to increase in direct proportion with the ageing population. Current treatments for OA help to mitigate the pain and suffering but fail to provide complete cure.

Conventional pharmacological/surgical treatments for articular cartilage injuries including arthroplasty for the replacement of damaged and diseased joints have decreased patient compliance as they rarely result in the full restoration of function. Especially, the young patients with a life potential beyond the lifetime of the prosthesis are likely to suffer more. Hence, there is a great necessity for the development of biological substitutes to aid restoration of damaged articular tissues with improved joint function. Adult cartilage has limited intrinsic self-healing capacity and cannot be spontaneously repaired due to the lack of vascular supply, poor matrix productivity and the low turnover of regenerated chondrocytes to the injured sites [2]. Use of autologous chondrocytes as a cell source for cartilage repair is being used for over a decade, and follow-up studies suggest that the treatment can provide real benefit, but the technique is limited to small lesions [3].

Understanding the underlying molecular mechanisms of cartilage formation, the biochemical composition and growth factors are important to aid cartilage differentiation/regeneration. Chondrogenesis in-vivo is initiated by sonic hedgehog signaling, which induces bone morphogenic proteins (BMPs) and directs mesenchymal stem cell differentiation into the chondrogenic lineage [4]. SRY (sex determining region Y)-box 9 (SOX9), a key transcription factor regulates cartilage formation and maintains the chondrocyte phenotype in the mature cartilage by activating the expression of several cartilage-specific genes, including collagen type II, alpha 1 (COL2A1) and aggrecan (ACAN). Several growth factors that promote chondrogenesis in vivo have also been demonstrated to promote chondrogenesis of mesenchymal stem cells (MSCs) in vitro [5].

Stem cell differentiation into cartilage and their transplantation offers a promising novel technique for the treatment of OA. There are diverse types of stem cells such as the human embryonic stem cells (ESCs), MSCs and the induced pluripotent stem cells (iPSCs). Pluripotent cells (ESCs, iPSCs) although are highly versatile, they can result in tumorigenesis upon in vivo transplantation [6]. In comparison, the multipotent MSCs is an attractive cell type given their self-renewal, increased proliferation, hypoimmunogenicity and differentiation potential [7]. MSCs can be obtained from various tissues including the bone marrow, adipose tissue, placenta and umbilical cords. Although the MSCs from bone marrow (BM-MSCs) are used widely for tissue engineering and regenerative medicine applications they have limited self-renewal ability as they are already an aged phenotype, being derived from adult tissues. Also, the cell harvesting procedure is invasive and painful with an additional risk of infection and donor site morbidity [8]. Unlike BM-MSCs the human umbilical cord mesenchymal stem cells (hUC-MSCs) are harvested from the discarded umbilical cord, which is usually considered as a medical waste. Importantly, the cell harvest is painless, available in abundance, have high proliferation (as they are very young compared to their adult counterpart), hypoimmunogenic and nontumorigenic [7, 9].

As such we in the present study evaluated the cartilage differentiation potential of the MSCs derived from the human umbilical cord Wharton's jelly (hWJSCs) in vitro and their characterization using histological and gene expression studies.

## Methodology

The ethical approval for the use of human umbilical cords following normal full-term delivery was obtained from the King Abdulaziz University (KAU) ethical committee [33-15/KAU].

### Derivation of hWJSCs

**Figure 1:**
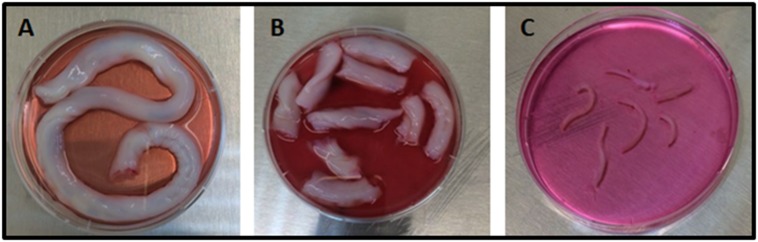
Representative image of the umbilical cord used for hWJSCs derivation. (A) The whole umbilical cord; (B) the umbilical cord sectioned into 2.5 cm long pieces (C) the umbilical cord blood vessels that were removed from two sectioned pieces of the umbilical cord.

The umbilical cords were collected following full term normal delivery at the King Abdulaziz University Hospital (KAUH), with informed patient consent and transferred in a sterile container containing DMEM with 2% antibiotics (penicillin, streptomycin) solution and processed within 2 - 4 hours. The umbilical cord (UC) was washed in phosphate buffered saline and cut into 2.5 cm pieces. The UC pieces were opened lengthwise and the blood vessels within were gently removed. We used enzymatic method [10] to isolate the cells from the umbilical cord (UC; Figure 1A-C). Briefly, the opened-up pieces of the UC were laid face down in a Petri dish containing an enzymatic cocktail of collagenases [Type-I and type II; 2 mg/mL] and hyaluronidase (100 IU) for 30 minutes at 37°C. Enzyme activity was neutralized with DMEM containing 10% fetal bovine serum (FBS), and the UC pieces were gently scraped with the blunt surface of a pair of curved forceps to separate the Wharton's jelly and the tethered cells into the medium. The medium with cells and matrix contents were centrifuged at 1000 rpm x 5 min and the supernatant discarded. The contents were washed twice with PBS and the resultant cell pellet was used for culture. DMEM high glucose medium supplemented with 10% FBS, 2mM Glutamax, 1% antibiotics solution (penicillin, streptomycin) and basic fibroblast growth factor (bFGF, 16 ng/ml) was used and the cells incubated under standard culture conditions of 37°C with 5% carbondioxide (CO2) in atmospheric air. Culture dishes were left undisturbed until cell growth was evident, except for fresh changes of media every 72h. Once confluent the cells were trypsinized and subcultured. Excess cells were cryopreserved until use in experiments and early passages (P3-P5) were used for the experiments.

### Cell morphology and proliferation

To evaluate the morphology and cell proliferation, the hWJSCs were plated at a seeding density of 2 x 104 cells/well in 24-well tissue culture plate and cultured for 24h, 48h and 72h under standard culture conditions. Cell morphology was obtained using phase contrast optics (Nikon instruments, Tokyo, Japan) and the cell proliferation was determined using MTT assay. Briefly, the MTT reagent (0.5 mg/mL) was added to the cells in culture and incubated for 4h until a purple precipitate was visible. The medium was then removed, and 100 mL of the detergent reagent added and incubated in the dark for 1h. Absorbance at 570nm was spectrophotometrically measured using a microplate ELISA reader (SpectraMax i3 Multi-Mode microplate reader, Molecular Devices, Sunnyvale, CA) with a reference wavelength of 650 nm.

### CD marker analysis

Derived cells were analysed for the presence of MSC related CD surface markers expression using fluorescent activated cell sorting (FACS) analysis as reported earlier [11]. Briefly, independent aliquots of cells (2 x 105) were treated with MSC isotype/phenotype cocktails (Miltenyi Biotec) or combination of individual antibodies (CD29, CD44; BD Pharmingen) to avoid interference of fluorochromes. The cells were incubated with respective primary antibody cocktails (1:10 dilution) for 15 min at 4°C. The cells were then washed with PBS solution containing 3% FBS and centrifuged (300 g x 5 min). The resultant pellet was resuspended in 500μl of 3% FBS before analysis using FACSAria III Instrument (BD BioSciences).

### hWJSCs differentiation into chondrocytes

The hWJSCs (2 x104 cells/well) were plated in a 24 well tissue culture plate and exposed to StemPro chondrogenic differentiation basal media (A100071-01, Thermo Fisher Scientific) fortified with chondrogenic supplements (StemPro Kit content) for up to 21 days with fresh changes of media every 72h. The control cells were treated only with chondrogenic basal media with no supplements. At the end of the differentiation period the cells were fixed in 4% formaldehyde solution (30 min), rinsed twice with PBS, stained with toluidine blue and analysed by light microscopy.

### Chondrocyte related gene expression analysis

The hWJSCs (2 x106 cells) were plated in T25 cm2 tissue culture flask and differentiated along the chondrocytic lineage as described above. Total RNA was isolated at the end of 21 days from both hWJSCs that were differentiated into chondrocytes and undifferentiated hWJSCs using Qiagen RNA extraction Kit (Invitrogen, Life Technologies). cDNA was synthesized with random hexamers using SuperScriptTM First strand synthesis system, which included prior DNase-I treatment step. Primers sequences (Table 1) were taken from earlier published studies [12]. Polymerase chain reaction analysis was performed with the ABI StepOne Plus Real-Time PCR System (Applied Biosystems, Foster City, CA) using SYBR Green and relative quantitation was performed using the comparative CT (2-ΔΔCT) method.

### Statistical Analysis

The differences observed between the control and treated hWJSCs following cell proliferation and gene expression assays were analysed using the Students t-test with the statistical package for Social Sciences (SPSS13). The results were expressed as mean ± standard error of the mean (SEM) from three different replicates for individual assays and a value of p < 0.05 was considered to be statistically significant.

## Results

### Morphology and proliferation of hWJSCs

**Figure 2:**
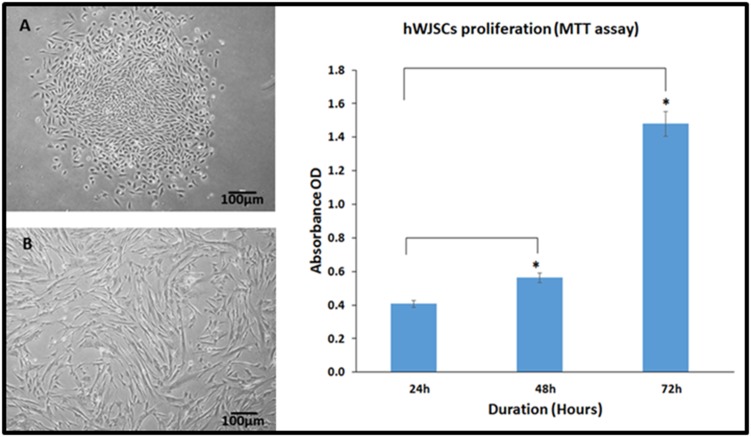
Phase contrast micrographs of human umbilical cord Wharton's Jelly stem cells (hWJSCs) showing the differences in morphology at various passages. (A) Early passage [P0] showing epithelioid cells with short fibroblastic morphology; (B) Late passage [P6] showing elongated fibroblast like cells. (C) hWJSCs proliferation (MTT assay) following culture for 24h, 48h and 72h. The values were expressed as mean ± SEM from minimium of 5 experimental replicates. Asterisk (*) indicate statistical significance at p<0.05 compared to control.

The hWJSCs were successfully derived from ten umbilical cords using the enzymatic method described in this study. The cells readily attached to the culture substratum and demonstrated active proliferation. Tissue debris and dead cells that existed at initial plating were gradually removed following media changes. Initial cultures of hWJSCs appeared as short fibroblasts and demonstrated active growth as a monolayer, which attained confluence within 5 to 7 days (Figure 2A). The hWJSCs in later passages appeared as long spindle shaped cells (Figure 2B). MTT assay demonstrated a mean increase in the numbers of hWJSCs with increase in culture period from 24h to 72h. There was a mean increase by 61.06% and 206.31% at 48h and 72h respectively, compared to 24h (Figure 2C). These mean increases in cell proliferation were statistically significant (P<0.05).

### CD marker analysis

**Figure 3:**
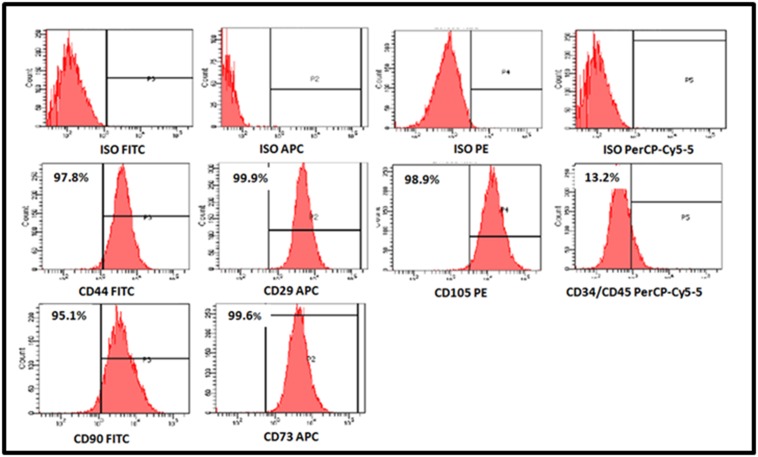
Representative histogram of the flow cytometry analysis (FACS) of the MSC related CD surface markers in human umbilical cord Wharton's Jelly stem cells (hWJSCs) from early passages. The hWJSCs demonstrated high positive expression of CD29, CD44, CD73, CD90 and CD105. The hWJSCs were negative for the haematopoietic stem cell markers CD34 and CD45.

The derived cells showed highly positive expression of MSC related CD surface markers in comparison with matched isotype controls (Figure 3). The percentage of MSC related CD surface markers were CD29 (99.9%), CD44 (97.8%), CD73 (99.6%), CD90 (95.1%) and CD105 (98.9%). These cells were negative for the haematopoietic related CD markers, namely CD34 and CD45 (Figure 3).

### hWJSCs differentiation into chondrocytes

**Figure 4:**
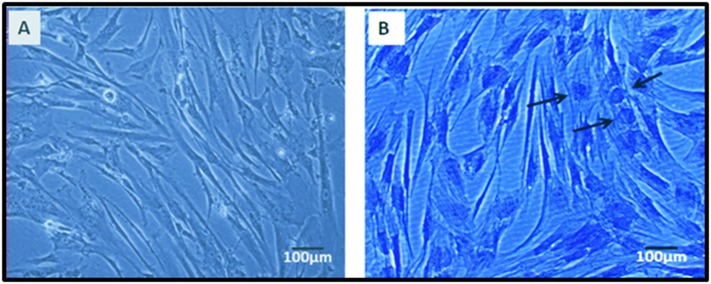
Toluidine blue histology. (A) hWJSCs cultured in chondrogenic basal medium for 21 days, showing normal spindle shaped cells (B) hWJSCs cultured in chondrogenic medium with supplements for 21 days showing positive staining and rounded chondrocyte like cells as indicated by black arrows. (Magnification 10X).

The hWJSCs cultured in chondrogenic differentiation media for 21 days demonstrated changes in cell morphology compared to the control. The hWJSCs lost their characteristic fibroblastic shape and attained globular shaped chondrocyte-like cells. These cells demonstrated positive staining with toluidine blue compared to the undifferentiated cells (Figure 4).

### Chondrocyte related gene expression analysis

**Figure 5:**
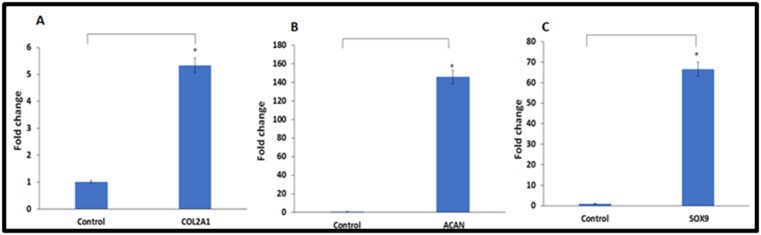
Quantitative real time gene expression analysis of the cartilage related genes in hWJSCs cultured in chondrogenic media for 21 days. Increased expression of (A) collagen, type II, alpha 1 [COL2A1], (B) aggrecan [ACAN] and (C) SRY (sex determining region Y)-box 9 [SOX9] was observed compared to controls. The values were expressed as mean ± SEM from 3 experimental replicates. Asterisk (*) indicate statistical significance at p<0.05 compared to control.

The hWJSCs cultured in chondrocyte differentiation media for 21 days demonstrated increased expression of some of the cartilage related genes namely collagen type 2 (COL2A1), aggrecan (ACAN) and SOX9 compared to the undifferentiated hWJSCs. The fold increases in COL2A1 (5 fold), ACAN (145 fold) and SOX9 (66 fold) were statistically significant compared to the undifferentiated control (Figure 5A-C).

## Discussion

Umbilical cords are usually discarded at birth even though they are an abundant source of stem cells. We successfully derived the stem cells that exist within the human umbilical cord Wharton's jelly (hWJSCs) using enzymatic method [10] and characterized their stemness and differentiation potential. The removal of umbilical blood vessels and the enzymatic digestion method employed in this study helps isolation of a relatively homogenous cell type unlike the explant culture method where the umbilical cords are dissected into small pieces without removal of the blood vessels. Although the explant culture method reduces the cell damage, as it does not involve enzymatic treatment, the main drawback is that primary cultures generated by this method are completely heterogeneous [13].

FACS analysis demonstrated positive expression of CD73, CD90, CD29, CD44 and CD105 surface markers (Figure 3) like earlier published studies [10, 14]. In addition, the derived hWJSCs demonstrated plastic adherence and the characteristic spindle shaped fibroblastic morphology; expressed the MSCs related surface markers expression and differentiation potential, thus satisfying the minimal criteria of the International Society for Cellular Therapy [15]. Unlike the MSCs isolated from adult tissues, the hWJSCs being relative young phenotype showed active cell proliferation with time. This was supported by earlier studies, which identified the doubling time of these hWJSCs to be much shorter [10, 11], thus it can support generation of sufficient cell numbers needed for regenerative medicine applications.

Cartilage is a specialized connective tissue that covers the ends of bones in joints. It mainly serves as a shock absorber, helps to prevent joint friction and protect the bone ends from traumatic injury or autoimmune processes, which are among the main causes of cartilage damage and degeneration [16]. Adult cartilage has limited intrinsic self-repair capacity and poor regeneration and therefore any longstanding cartilage damage results in OA. Spontaneous repair of the damaged cartilage largely remains impossible due to the absence of vascular supply, poor matrix productivity and the low provision of regenerated chondrocytes to the injured sites [17].

Differentiation of hWJSCs into chondrocytes as in the present study offers renewed hope for cartilage repair/regeneration. Although the differentiated cells did not exhibit the characteristic chondrocytic morphology, their positive staining with toluidine blue (Figure 4) compared to undifferentiated hWJSCs indicated that hWJSCs were indeed undergoing differentiation along the chondrocytic lineage. Earlier studies by different research groups have demonstrated the cartilage differentiation potential of perinatal stem cells including the WJSCs [18]. Furthermore, use of nanomaterials to aid chondrogenic differentiation of WJSCs [19] can accelerate the cartilage tissue engineering and clinical translational prospects. However, further in-depth confirmation of chondrocyte differentiation following pellet or micromass cultures and additional histological staining with alcian blue and/or safronin O will support translational initiative and will be considered in future studies.

Increased expression of cartilage related genes in this study namely the COL2A1, ACAN and SOX9 (Figure 5) further indicate that the hWJSCs cultured in chondrogenic media for 21 days were effectively undergoing differentiation along the chondrocytic lineage. Demonstration of positive immunohistochemical staining for COL2A1 and ACAN [20]; increased production of hyaluronic acid and sulfated glycosaminoglycans (GAGs) using biochemical assays [12]; as well as the expression of SOX9, COL2A1 and ACAN genes [12] in the differentiated hWJSCs indicate that these cells can be successfully used for cartilage repair/regeneration.

## Conclusions

The hWJSCs isolated from within the human umbilical cord was successfully differentiated into chondrocytes. These cells are relatively young, can be harvested in abundance and has several other advantages especially being non-tumorigenic compared to other MSC types [21]. Moreover, their unique features of low immunogenicity and their potential to induce immune tolerance in the host justify the efforts for their use in osteoarthritis, rheumatoid arthritis and other disease settings. The hWJSCs is therefore a promising cell type for cartilage repair/regeneration.

## Conflict of interest

All authors have no conflict of interests

## Figures and Tables

**Table 1:** The genes and primer sequences used for quantitative real time PCR. F: Forward primer; R: Reverse primer. GAPDH: Glyceraldehyde 3-phosphate dehydrogenase; SOX9: SRY (sex determining region Y)-box 9; ACAN: Aggrecan; COL2A1: Collagen, type II, alpha 1

Gene name	Sequence primer
GAPDH	F: 5'- GCACCGTCAAGGCTGAGAAC -3'
	R: 5'- GGATCTCGCTCCTGGAAGATG -3'
SOX9	F: 5'- GTACCCGCACTTGCACAAC -3'
	R: 5'- TCTTCCTGGTGGTGGGCCTAATG -3'
ACAN	F: 5'- AGA CTT GGT GGG GTC AG-3'
	R: 5'- TGT ATC ACC CCT TTG TAG -3'
COL2A1	F: 5'- GTG ACA AAG GAG AGG CTG GA -3'
	R: 5'- CAG GAA GAC CGG GAT CTC C -3'
